# Open science and data sharing in cognitive neuroscience with MouseBytes and MouseBytes+

**DOI:** 10.1038/s41597-023-02106-1

**Published:** 2023-04-14

**Authors:** Sara Memar, Eric Jiang, Vania F. Prado, Lisa M. Saksida, Timothy J. Bussey, Marco A. M. Prado

**Affiliations:** 1 BrainsCAN, The University of Western Ontario, London, Ontario N6A 3K7 Canada; 2 Robarts Research Institute, The University of Western Ontario, London, Ontario N6A 3K7 Canada; 3 Department of Physiology and Pharmacology, The University of Western Ontario, London, Ontario N6A 3K7 Canada; 4 Department of Anatomy and Cell Biology, The University of Western Ontario, London, Ontario N6A 3K7 Canada

**Keywords:** Attention, Motivation, Dynamical systems, Cognitive ageing

## Abstract

Open access to rodent cognitive data has lagged behind the rapid generation of large open-access datasets in other areas of neuroscience, such as neuroimaging and genomics. One contributing factor has been the absence of uniform standardization in experiments and data output, an issue that has particularly plagued studies in animal models. Touchscreen-automated cognitive testing of animal models allows standardized outputs that are compatible with open-access sharing. Touchscreen datasets can be combined with different neuro-technologies such as fiber photometry, miniscopes, optogenetics, and MRI to evaluate the relationship between neural activity and behavior. Here we describe a platform that allows deposition of these data into an open-access repository. This platform, called MouseBytes, is a web-based repository that enables researchers to store, share, visualize, and analyze cognitive data. Here we present the architecture, structure, and the essential infrastructure behind MouseBytes. In addition, we describe MouseBytes+, a database that allows data from complementary neuro-technologies such as imaging and photometry to be easily integrated with behavioral data in MouseBytes to support multi-modal behavioral analysis.

## Introduction

Neuroscience data are often rich, and the same set of data can be used efficiently to answer a number of research questions of interest to disparate groups of researchers. However, sharing data amongst researchers so that such questions can be answered has been challenging, even within a single lab or between collaborators. Such issues severely limit the re-usability of data outside of the context of the original experiment. Lack of metadata required for data interpretation and different structures for storage have made data reproducibility and comparison even more complicated. Open Science has played a fundamental role in addressing such limitations by emphasizing public communication throughout a project, from idea generation to post-publication discussion^1^. However, open communication and making research data generally ‘accessible’ in non-standard or structured formats is not sufficient: the increasing volume, complexity and speed of data creation has caused an urgent need to enhance the infrastructure supporting the reuse of research data. FAIR Data Principles, which were initially proposed in 2016^2^ can help to improve the re-usability of data and ensure that the findings and data behind research outcomes are findable, accessible, interoperable, and reusable^3^. The value and importance of Open Science and FAIR principles has led to the development of data sharing platforms across neuroscience disciplines, particularly in areas such as neuroimaging and genomics, and has facilitated a paradigm shift in the way we are now able to analyze, share, and re-use vast amounts of data from multiple laboratories^4–8^. For example, a combination of anatomical, functional, and diffusion magnetic resonance imaging (MRI) datasets from laboratories throughout the world was aggregated in the PRIME Data Exchange platform (https://prime-re.github.io/data_sharing.html#prime-de), an Open Science resource for the neuroimaging community that facilitates the mapping of the non-human primate connectome^5^. A broad range of genomic analyses about specific phenotypes as well as genome-wide properties and principles can be found in ENCODE (https://www.encodeproject.org/) genomic repository^6^. These platforms, repositories, and analytic tools streamline reproducibility and data availability and empower novel investigations and collaborations on a scale never seen before.

Assessing behavior in animal models is a critical component in our understanding of normal brain functions, as well as to unveil new ways of treating brain dysfunctions in neurodegenerative and neuropsychiatric diseases^9–12^. Unfortunately, the level of integration and sharing in areas of neuroscience like neuroimaging and genomics has not yet been fully adopted in the analysis of behavioral data in animal models. In particular, the lack of automation and standardized outputs in conventional behavioral assessment of rodents, the main animal group used in pre-clinical research, can hinder the use of shared data.

Classical behavioral experiments have protocols that vary between different laboratories and even within the same laboratory, and do not allow for sharing of structured and well-organized datasets. Hence, current data-sharing platforms for conventional behavioral data lack information on standardization decreasing the potential for re-use of data. The development of computerized and automated behavioral testing is a game-changing opportunity in cognitive behavioral data collection and analysis^13–17^. Touchscreen technology^18^ has enabled the automation of multiple high-level cognitive tests and the generation of data in digital formats that lend themselves to deposition in FAIR-compliant data repositories. This technology has now been used in over 400 labs worldwide for the deep phenotyping of cognitive function in animal models^19^. Moreover, touchscreens tests can be easily synchronized with neuro-technologies such as fiber photometry, miniscopes, and optogenetics, to evaluate the cellular, neurochemical and circuit basis of behaviour^20–24^. Touchscreens are either commercially available or can be assembled using open sources (https://edspace.american.edu/openbehavior/open-source-tools/behavior-apparatus/), providing a platform where multiple labs can easily standardize behavior using similar platforms.

To leverage the development of such a highly reproducible and standardized cognitive assessment tool and their increased use in multiple research sites, we have developed MouseBytes (https://mousebytes.ca/home), a software and web application that enables researchers across the globe to pre-process, run automated quality control scripts, visualize, and analyze their data alone or alongside the stored data of other researchers. This platform is a fundamental step towards facilitating data availability, transparency, and reproducibility.

High-level introduction of MouseBytes and the essence of the domain-specific data repository for manipulating touchscreen-based rodent cognitive behavioral data was first presented in 2019^25^. This study was mainly focused on the application of MouseBytes for curating and depositing the largest age-dependent dataset collected and analyzed to investigate novel sex and genetic background related to cognitive domains in a multi-site cohort of Alzheimer’s disease (AD) model mice.

Here we present the design, architecture, infrastructure, and functionalities in MouseBytes and how to navigate and use them for data curation. Such functionalities include creating and managing experiments, sub-experiments, and animals and their corresponding metadata, personalized visualization for the private data such that only the owner of data can browse the graphs, the dashboard page for high level overview of MouseBytes data, and the technologies used and implemented for developing this application. Moreover, MouseBytes+ (https://mousebytes.ca/comp-search), which incorporates complementary datasets obtained using multiple new technologies, such as fibre photometry, optogenetics, miniscopes, and different imaging modalities, is introduced and explained. We emphasize in particular the importance of the functionalities implemented in MouseBytes and MouseBytes+ to make these platforms aligned with required guidelines, desired characteristics, and recommendations determined to facilitate data management and sharing^3,26–28^.

## Results

### Community use of MouseBytes

**Fig. 1:**
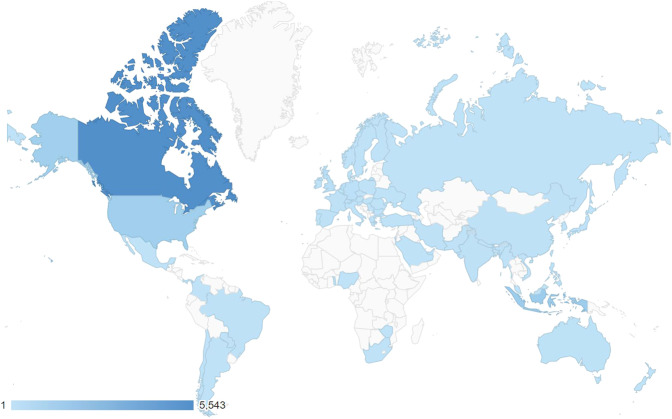
Countries where users browsed MouseBytes.

To date, the data from over 3,000 individual mice has been deposited in MouseBytes, either as public or private datasets. We encourage authors to make their datasets publicly available and linked to their publications using the Digital Object Identifier (DOI) for published manuscripts. These deposited data represent a broad range of animal disease models, cognitive tasks, age, and sex providing a coherent and comprehensive platform to evaluate how cognitive measures (e.g., attention, memory and behavioral flexibility) are affected in different rodent models of diseases. Based on the Google Analytics https://en.wikipedia.org/wiki/Google_Analytics), the MouseBytes web pages have been viewed over 30,000 times since February 2019. There have been 13,000 homepage visits from 4,600 individual users. This provides an idea of the number of people that would have access to datasets, as this function does not require users to be logged in. Figure 1 shows the countries of users who accessed MouseBytes. Canada, US, and Australia had the most visitors, which reflects the large number of laboratories using touchscreen technology in these countries. Datasets have been downloaded 868 times. Moreover, one of the important features of MouseBytes is the ability of users to create unique hyperlinks that are used to download specific datasets. To date, 1200 unique links were generated for access of datasets since June 2020.

The general overview of data in MouseBytes is available on the dashboard (https://mousebytes.ca/mb-dashboard) which aggregates the key metrics of the platform in one place. On this page, any website visitor can view a variety of information depicting the various cognitive behavioral tasks in the MouseBytes database and various high-level statistics related to them, including number of datasets, data points, and animals, as well as breakdowns for age and sex. The dashboard also allows users to interactively filter the presented data and graphs based on these parameters (task, age, sex, genotype) thus enabling a comprehensive meta-analysis of subjects and trends in touchscreen research. From the dashboard, one can derive some useful statistics for datasets, cognitive tasks currently available, and other experimental parameters in MouseBytes. There are currently 88 datasets that cover 10 cognitive tasks in the system. A large set of datasets (57) are set to private, suggesting these are ongoing experiments, whereas 31 datasets are public, and many of them associated with published papers via their DOIs^25,29^. Currently, a total of 13 laboratories, with 17 Principal investigators and 69 users, have registered with MouseBytes to upload datasets. Over 90% of all the data points in MouseBytes are from 4 cognitive tasks: 5-CSRTT (5 C), Pairwise Visual Discrimination (PVD), Paired-Associates Learning (PAL), and Image Continuous Performance Task (CPT), with over 40,000 data points for both 5 C and PVD, over 25,000 for PAL, and over 15,000 for CPT. In each of these tasks, the most frequently studied rodent models are amyloid models based on expression of mutant amyloid precursor protein (APP), presenilin and the phosphoprotein tau, followed by TDP-43 mutant mouse models (a model of ALS and frontotemporal dementia). Younger mice predominate in the platform, with about half of the data points originating from mice between 3 and 6 months of age in the 5 C, PD, and PAL tasks, and the number of data points decreasing with each age group above that (with CPT having a much more even distribution of data points for ages from 3 to 13 months). In 5 C, PD, and PAL, data points from male mice outnumber that of female mice by a ratio of about 6:4 while CPT datasets have about an equal number of male and female mouse data points.

### Touchscreen technology

**Fig. 2:**
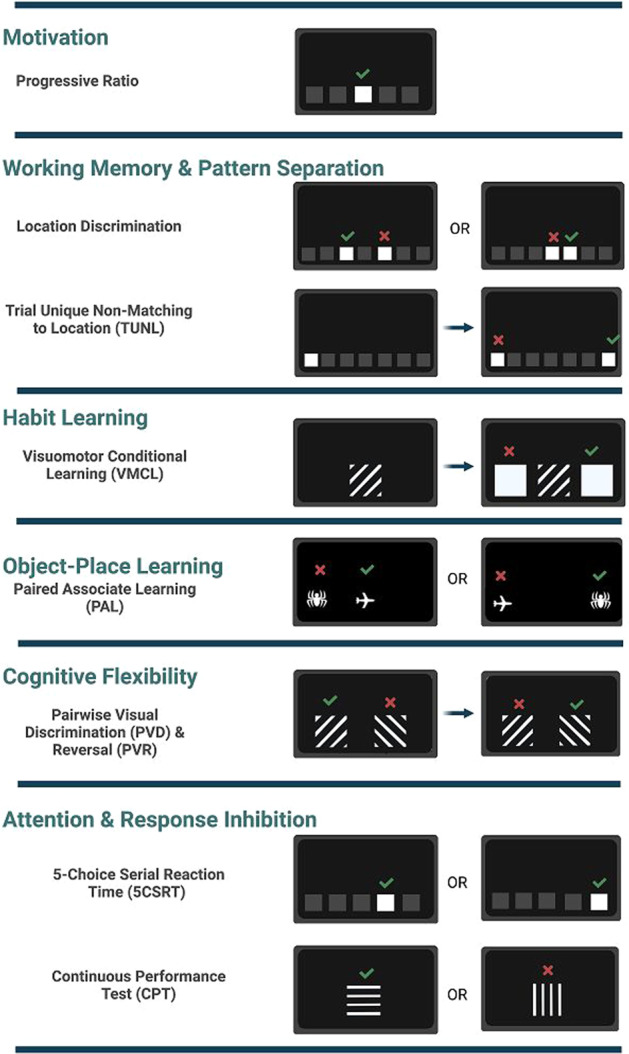
Touchscreen tasks.

First described in 1994^30^, touchscreen tasks are highly similar, and in some cases identical, to human cognitive tests^31,32^. There are a variety of novel cognitive tasks developed in the touchscreen technology to explore cognition in rodents; for example, paired-associates learning (PAL)^33,34^, the location discrimination (LD) test of pattern separation^35^, trial-unique nonmatching-to-location (TUNL), the rodent continuous performance task (rCPT)^36^, and 5-Choice serial reaction time task (5-CSRTT) have been used to investigate reward learning, memory, perceptual discrimination, object-place associative learning, attention, impulsivity, compulsivity, extinction, simple Pavlovian conditioning, and other constructs in rodents^31^. As shown in Fig. 2, for example, the screen was divided into five partitions in the 5-choice task, and the mouse responds by touching the flashes of light displayed randomly in one of the five windows on the touchscreen chamber in which every correct response is followed by reward. The key advantage of this system is the consistency of tasks in terms of stimuli, required responses on the part of the subject and feedback which facilitates the comparison of the performance across the battery of the tasks. Furthermore, the automation of high-level cognitive testing allows response latencies to be measured with milli-second accuracy and helps with the sensitivity of the tasks for exploring cognitive impairments^37^.

### MouseBytes functionalities

**Fig. 3:**
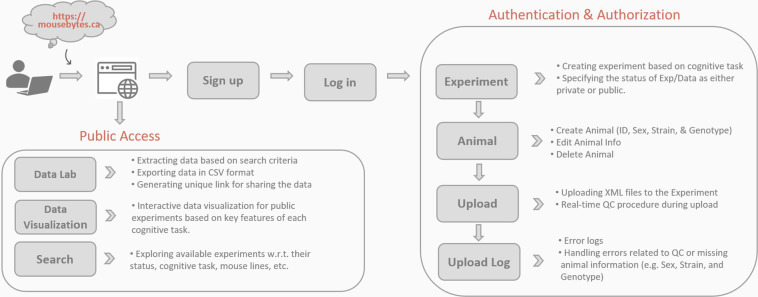
MouseBytes services.

MouseBytes includes many different functionalities for researchers to facilitate cognitive behavioral data collection, analysis, and sharing. Figure 3 indicates the web pages developed in MouseBytes for different functionalities. Each of these aims to ensure that MouseBytes is compatible with the FAIR principles and is a comprehensive platform for all the fundamental needs of researchers in the field. This includes: allowing users to create experiments and sub-experiments and designate appropriate metadata related to them for referencing; allowing users to upload data files associated with their experiments; allowing users to create complementary data repositories associated with these experiments that do not take the form of touchscreen data (such as fibre photometry data or analysis files), allowing for the extraction of public datasets in the system for third-party analysis, and visualizing the data through graphs that can be filtered for various experimental features (such as the experiment name, sex, strain, etc.). Some services, such as Data Lab (https://mousebytes.ca/data-extraction), Data Visualization (https://mousebytes.ca/data-visualization) and Search (https://mousebytes.ca/search-experiment), are open to the public, while other services require authentication and authorization via log in credentials. The public services facilitate the open access and presentation of data critical to Open Science by being available for all users, while the user-only services are designed to be user-friendly and convenient for the researcher, to incentivize them to publish their data on the platform.

#### Data indexing and metadata

Metadata play an essential role for maintaining the historical records of datasets, and they are mainly employed to summarize the key information about research data and to facilitate accessing, identifying, tracking, and downloading data for reuse. In MouseBytes, metadata searching allows users to consistently organize, upload, and revise their data through the required metadata associated with the different elements of the experimental research. The majority of the metadata in this process are collected through drop-down menus to provide consistent representation of the data.

Unlike some services in MouseBytes which are open to the public, credentials are required for uploading datasets to the system. Users need to sign up and sign into the system in order to create an *Experiment* based on a cognitive task and add the data to the experiment. The metadata associated with each experiment are title, start and end dates of experiment, cognitive task, species (e.g. mouse, rat, marmoset), PI (principal investigator) and institution, status of the data (private or public), battery of tasks to indicate which cognitive task(s) are followed by the current data for the same subjects, DOI (Digital Object Identifier) if the data was already published, and experiment description. Since animals may be tested with different conditions, several *Sub-experiments* can be added to the experiment to link all the data to the associated variables of the experiment. In this step, age, intervention, light cycle, housing information, and the types of the stimuli are collected as the required metadata for reproducibility. The features associated with each animal are added to the experiment using the *Animal* service to collect animal ID, sex, strain, and genotype. Users can then upload the data to the corresponding experiment and sub-experiment using the *Upload* service and check their data through a quality control procedure. There is also a dashboard in the *Upload Log* service listing the files flagged by the quality control procedure and enabling the user to handle errors. For instance, if the animal information is missing for the uploaded file, it will be flagged and listed in the dashboard, allowing the user to resolve this issue by adding the missing information. Moreover, the quality control procedure also checks for file duplication and ensures the file is compatible with the given experiment and cognitive task. Quality control documents for all the cognitive tasks in the MouseBytes are available in the guidelines page and *Quality Control Rules* (https://mousebytes.ca/guideline) tab. However, cognitive data that cannot pass quality control due to a different protocol can still be uploaded and shared through MouseBytes+.

#### SEARCH

**Fig. 4:**
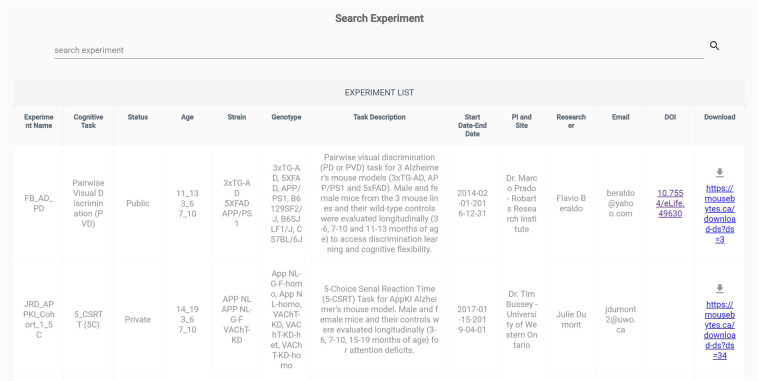
MouseBytes datasets search.

All of the key metadata associated with each experiment/dataset can be found in the *Search* page (https://mousebytes.ca/search-experiment) which works as a landing page where all the metadata for each dataset are summarized in a table along with the DOI generated from DataCite (https://datacite.org/https://datacite.org/) as a persistent identifier for each public dataset. All datasets in MouseBytes and MouseBytes+ are assigned a DOI when they become public. DataCite is a global non-profit organization serving the research community with DOI and metadata registration for digital objects like datasets and other scholarly research outputs to make them accessible and citable. As illustrated in Fig. 4, there is a search section, and if the user types “public” and hits enter, all the public experiments are filtered. In addition to the status of the experiments (e.g., public or private), the list of experiments can be filtered based on task, age, strain, and genotype. Furthermore, a unique and persistent link identifier associated with each dataset is available in the last column of the table in this page. So, all the data related to an experiment for a cognitive task along with its metadata can be obtained via the persistent unique link in CSV format.

#### DATA LAB

Data lab is part of the main data storage for MouseBytes where users can query the database based on different search criteria, enabling users to connect to the database and explore the data in a user-friendly manner. The status of each dataset/experiment can be set to either private or public in MouseBytes. Data with public status can be extracted and shared under the Open Licence *(Creative Common 0 (*https://creativecommons.org/publicdomain/zero/1.0/*))*, allowing other users to reuse, re-analyse and share the data freely without any restriction as long as the source is cited. On the other hand, access to the private datasets is limited to the owner of the data or whomever the owner decides to share the data with. Search queries are defined by drop-down menus and include, but are not limited to, criteria such as cognitive task, experiment title, various features associated with the selected cognitive task, PI, site, intervention, animal age, sex, strain, and genotype. These critical features of experimental design and datasets can then be used to identify datasets of interest. Thus, open datasets can be queried and extracted using the *Data Lab* (https://mousebytes.ca/data-extraction). The description of the terms used in the task and session drop down menus were provided via the tooltips once users hover the mouse over any values. To further inform users, there is a Glossary in the *guidelines* (https://mousebytes.ca/guideline) page covering the definitions of the tasks, features, and all acronyms used in the system.

Users have the option to extract the data trial-by trial or aggregated based on either mean, standard error, count, or sum. Data extraction in MouseBytes is subject to a confirmation of *terms of services* (https://mousebytes.ca/terms). A unique link can be generated for the queried data allowing for quick and convenient access to the extracted data in the future. The owner of the data can also use the unique link to share the data with other investigators, even if the experiment status is private. Furthermore, these links can be added to publications for directing readers to the corresponding data in MouseBytes that are associated with specific figures. For instance, in a recent study^25^, multiple unique URLs were created and linked to each of the figure panels in which key features of the experiment were compared. Conversely, the DOI of the published manuscript can be linked to the corresponding experiment(s) to facilitate their retrieval. The extracted data can be also exported into CSV format for additional analysis. This feature of MouseBytes provides authors, readers, journals and funding agencies with an easy way to identify primary datasets for each figure or figure panel, increasing transparency and helping to increase reproducibility.

#### Data visualization

**Fig. 5:**
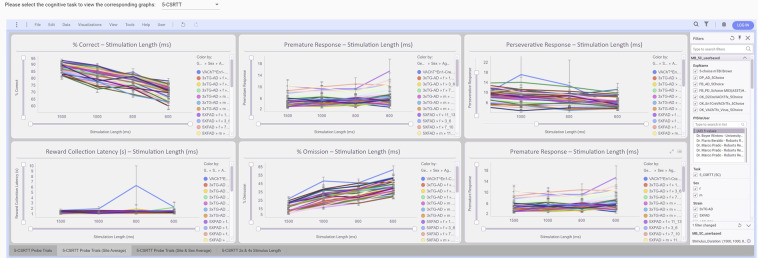
Graphs of 5-choice key features at different stimulus duration.

Data visualization enables users to easily browse and compare datasets linked to a cognitive task in an interactive way across different features such as site, age, sex, strain, genotype, etc. MouseBytes hosts an interactive and accessible platform to browse and understand the data and their analysis through the *Data Visualization* (https://mousebytes.ca/data-visualization) service based on TIBCO Spotfire Analytics (https://www.tibco.com/products/tibco-spotfire). Spotfire is a powerful analytical platform which can be linked to different data sources to transform data into valuable insights via an effective dashboard and interactive visualization. MouseBytes Data, hosted in Microsoft SQL Server (https://www.microsoft.com/en-ca/sql-server/sql-server-downloads), are linked to this tool as a data source. The key features associated with each cognitive task are analyzed and visualized in Spotfire, and the generated graphs are presented in an integrated data visualization page in MouseBytes through API calls. In the *Data Visualization* (https://mousebytes.ca/data-visualization) service, users first select the cognitive task in the drop-down menu, and the corresponding graphs associated with the key features of the task are displayed. There is also a filtering panel on the right side of the page which enables users to filter data based on metadata such as site, mouse strain, genotype, sex, age, etc., and the graphs are automatically updated according to the selections. For instance, Fig. 5 shows the graphs related to the key features of 5-choice cognitive task. The primary metric for this task is %Correct, and other features are secondary features obtained at different stimulus duration for assessing attention. MouseBytes visualization not only depicts the analyzed data for the public datasets, but private datasets can now be visualized by the owner provided that the owner of the private dataset is logged into the system. This enables the users to compare and validate new data and experimental design against published data as soon as they upload them to the system.

### MouseBytes+

**Fig. 6:**
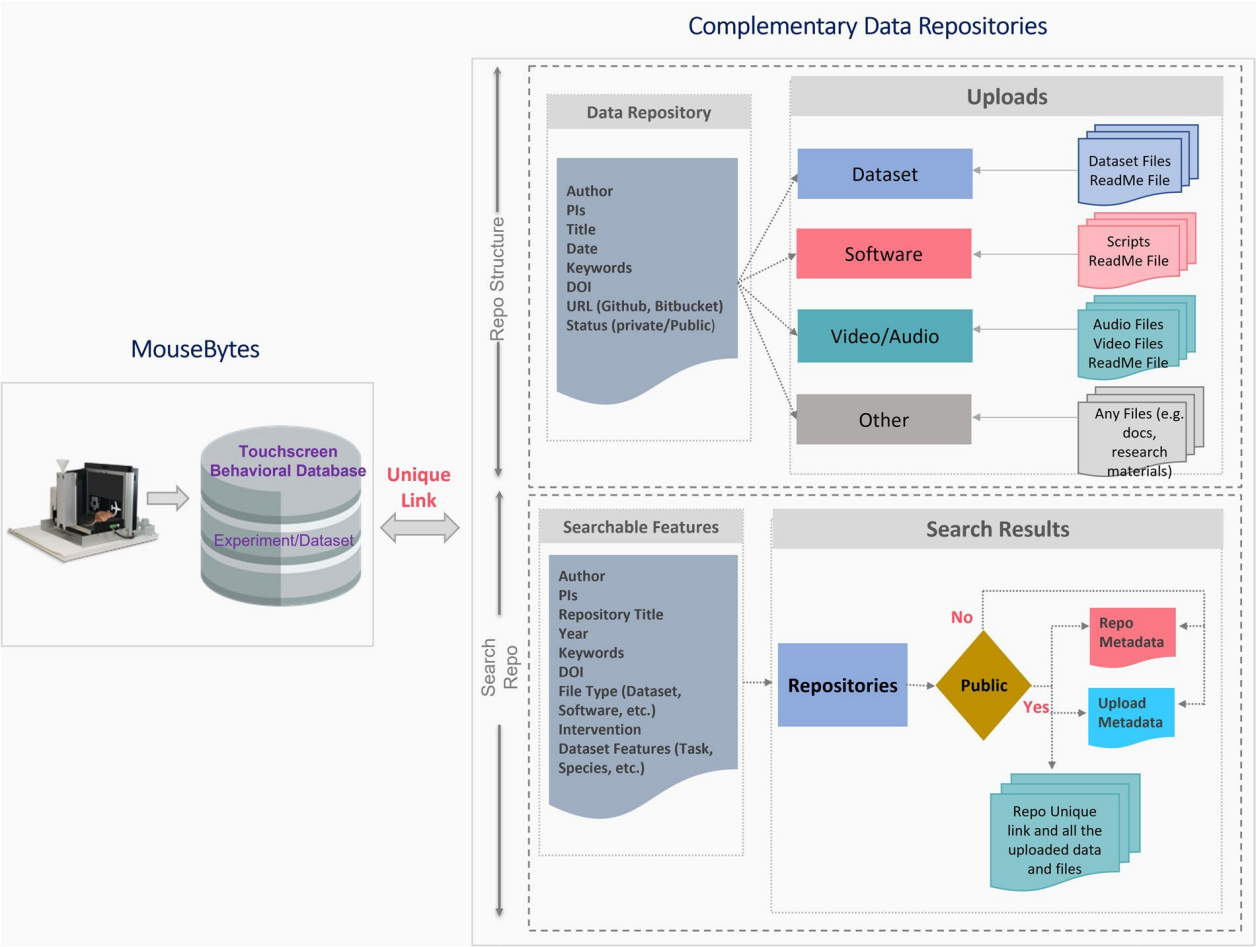
MouseBytes+ components.

**Fig. 7:**
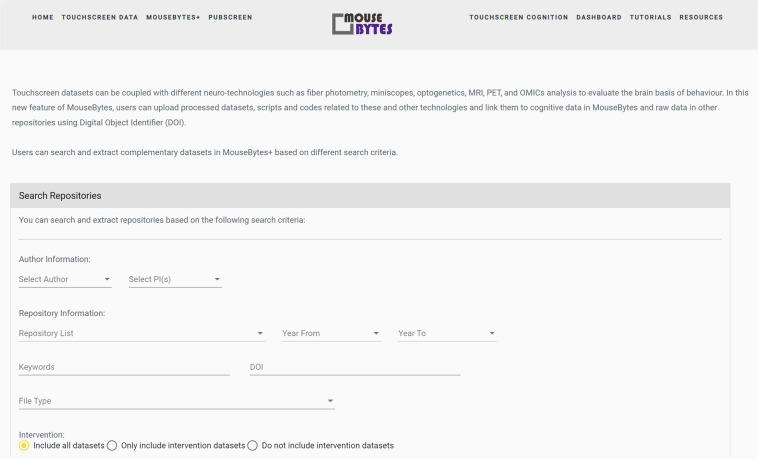
MouseBytes+ search repositories.

One fundamental challenge faced by neuroscientists is that the capacity to collect data exceeds the ability to integrate datasets across modalities to understand links between brain and behavior. It is useful to be able to access datasets that complement behavior and correlate them to cognitive function. For example, whole brain imaging can inform about structural and connectivity changes, whereas electrophysiological recording can add information about activity of ensembles of neurons. Importantly, *in vivo* recordings of both neurochemical activities and individual neurons have become common in freely-behaving mice, adding a layer of complexity to behavioral datasets. To accommodate these needs, we created a complementary database (MouseBytes+ (https://mousebytes.ca/comp-search)), that is structured and uses as a nexus cognitive datasets, but allows the flexibility for authors to upload complementary datasets that can be correlated with behavioral analysis. To illustrate the need for storing datasets supplementary to cognitive behavioral data, we can consider the measurement of *in vivo* neural activity. *In vivo* electrophysiology has been considered the gold standard technique to investigate neural dynamics in freely behaving animals. However, over the last decade, genetically encoded calcium indicators (GECIs), voltage indicators and neurotransmitter biosensors have been widely adopted as versatile tools to monitor cell-type-specific population activity *in vivo*. Touchscreens can be synchronized with neuro-technologies such as fiber photometry, miniscopes, and also with technologies for manipulation of cells, such as optogenetics or chemogenetics. Hence, it is important to provide the means to link these different data modalities in a single integrated platform, ranging from behaviour to brain structure and function, and neural activities to support comprehensive multi-modal data analysis. The *MouseBytes+* component in MouseBytes achieves this by allowing users to store, extract, and share additional files related to their touchscreen experimental data, including processed datasets, metadata, and supplementary analytical methods related to a variety of neuro-technologies. This can range from fibre photometry recordings for cellular activity or neurochemical signalling, miniscopes for cellular activity, electrophysiology, magnetic resonance imaging (MRI) positron emission tomography (PET) and other imaging or OMICs modalities that relate to the animals being studied. It is also flexible enough to allow the upload of any other file tangential to the experiment, including software files associated with the experimental process, audio/video files, procedural documents and READ-ME files. These complementary files can be linked to the relevant cognitive behavioral data in MouseBytes and raw data in other repositories via the DOI or a unique link generated automatically by the system. Figure 6 shows an overview of MouseBytes+. For each repository entry, users enter a series of searchable metadata features including Author, PI, Title, Date, Keywords, DOI, URL links for any additional files associated with the data, and Status (public or private). Within each entry, users may then create any number of different uploads, which allows the users to organize their files based on their file types. These types include datasets (with separate types for Touchscreen, Fiber Photometry, MRI Imaging, and Miniscope Data), Software, Video/Audio, or Other for miscellaneous data. Uploads are then named by the user and given a description and other metadata features in case of datasets, including as Cognitive Task, Species, Sex, Strain, etc. Finally, users add the corresponding files to each upload. MouseBytes also enables all visitors of the website to search these complementary data entries. As shown in Fig. 7, users may select various features they wish to select for including Author, PIs, Repository Title, File Type, and in the case of dataset files, key features associated with the data listed. The results of the search present all of the corresponding repositories and uploads in the database, a list of all of its associated metadata features, and a list of any MouseBytes experiments (i.e. touchscreen datasets) that the repository is linked to. Finally, if the repository has a public status, the search result will include the list of files within each upload and allow the visitor to download any or all of the files within.

## Discussion

Desirable characteristics of data repositories were outlined in National Institutes of Health (NIH) scientific data management and sharing policy^28^. The platforms (i.e., MouseBytes and MouseBytes+) we have developed are compatible with such characteristics by providing unique persistent identifier for each dataset along with metadata, free and easy access to the public datasets based on the *terms of service* (https://mousebytes.ca/terms), and quality control rules for depositing complete and accurate data into the system. In terms of long-term sustainability, two on-premises servers (i.e., Linux-based and Windows-based servers) have been maintained by Western Technology Service (WTS) at the University of Western Ontario. Moreover, we are working with a scientific consulting board to provide guidance for Touchscreen datasets standardization and governance practices. Data in these platforms are under Secure Sockets Layer (SSL) certificate to ensure the confidentiality and integrity of data-in-transit.

Here we describe the architecture, functionalities and use of MouseBytes, a FAIR-compliant platform for storing, visualizing, and analyzing structured cognitive datasets. Included within the MouseBytes platform is MouseBytes+, which houses electrophysiological and photometric data, MRI, PET and miniscope imaging data that can be integrated with data from cognitive testing. Integrating these complementary datasets is critical for understanding how brain changes give rise to complex cognition.

**Fig. 8:**
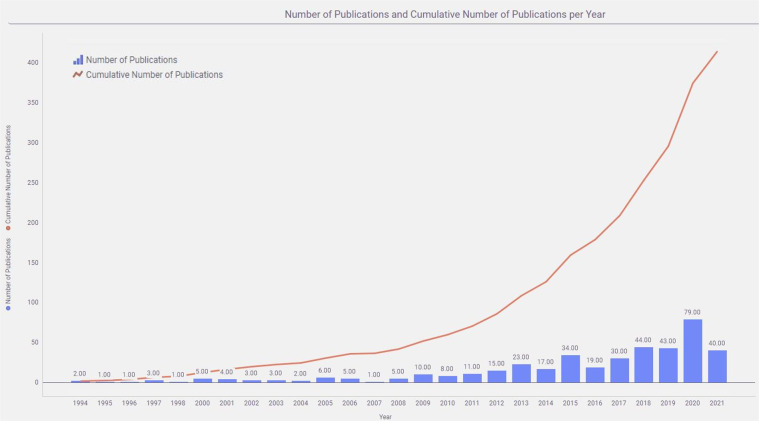
Number of Touchscreen-based publications and the cumulative number of publications per year.

Cognitive testing using touchscreen technology with animal models, especially rodents, is currently used in over 400 research groups at over 200 research institutes in at least 26 countries^19^. Such popularity has resulted in prolific research outcomes that are increasing exponentially^38–49^. As Fig. 8 indicates, over 400 manuscripts have been published since 1994 using touchscreen technology to evaluate different cognitive domains. Of these papers, an impressive 79 were published in 2020 alone. The widespread adoption of this form of cognitive testing, with its automation, potential standardized outputs, and high level of reproducibility affords the community with a valuable platform for understanding relationships between brain and behaviour. The challenge is now to make sure all these generated data can become available for reuse.

The International Neuroinformatics Coordinating Facility (INCF) has provided a set of recommendations and associated criteria for developing a FAIR compliant repository or scientific gateway for the neuroscience community^27^. One of the key characteristics of Open Science which is recommended by the INCF is user engagement and community building. Hence, an online touchscreen community, (Touchscreen Cognition (https://touchscreencognition.org/)) was developed to better serve the diverse users of touchscreen technology embracing the goals and values of Open Science. Task-specific standard operating procedures (SOPs), training courses, events, and videos tutorials explaining steps for using touchscreens can be found there. There is also an online forum enabling users to discuss challenges and share their experiences. MouseBytes plays an important role within this community. Whereas the goal of Touchscreen Cognition is knowledge dissemination and open communication, MouseBytes is focused on data sharing and analysis. The integration of these two tools facilitates communication through the user engagement forum in Touchscreen Cognition. Furthermore, to be aligned with the recommendations developed by INCF, guidelines (https://mousebytes.ca/guideline) and video tutorials (https://mousebytes.ca/video-tutorial) for both MouseBytes and MouseBytes+ are provided to streamline the functionalities in these applications and help users learn how to process their data in an easy and effective manner. There is also a Glossary in the *guidelines* page explaining the definitions of the tasks, features, and all acronyms used in the system.

MouseBytes, like other Open Science platforms, has brought many benefits to the cognitive neuroscience community^50–52^. Robust experimental design is a key for reproducibility. MouseBytes and MouseBytes+ provide a way to compare cognitive data and related brain activity across different ages, mouse strains, sex, and laboratories, allowing researchers to assess robustness without the need to perform new experiments. For example, it allows the user to analyze their raw behavioral data alongside other researchers’ stored data, giving the user quick feedback about expected variance across laboratories.

Moreover, the MouseBytes platform allows the emergence of scientific consortia, without the need to have multiple labs formally being part of the consortium or being funded to be part of consortium. This contrasts with other similar platforms such as the International Brain Laboratory^53,54^, which requires specific funding schemes and membership to participate in their consortium for behavioral assessments in head-fixed mice.

Most journals and funding organizations have recently required that data become available after publication as one of their conditions for accepting manuscripts or funding grants. Hence, MouseBytes can significantly assist the touchscreen research community by providing a way by which structured raw data can be accessed and shared. We foresee MouseBytes and MouseBytes+ as major steps towards increasing the availability of datasets, including negative results, that can serve to fulfill requirements by journals and data management requirements for grants. MouseBytes can also improve equity by providing labs and researchers with access to curated cognitive behavioral data at no cost.

Beyond answering scientific questions, such multi-modal data could be employed to answer questions about how these multiple levels of mechanisms interact. The neuroinformatics framework described here can be easily adapted by other research groups using complementary data-streams to expand work across disorders and high-dimensional data types. Working in the framework of Open Science, this fundamental innovation will democratize access to complex datasets, giving researchers powerful tools to integrate multi-dimensional, multi-modality datasets and thus remove a substantial barrier to advancing the understanding of neural mechanisms of brain function in health and disease.

## Method

**Fig. 9:**
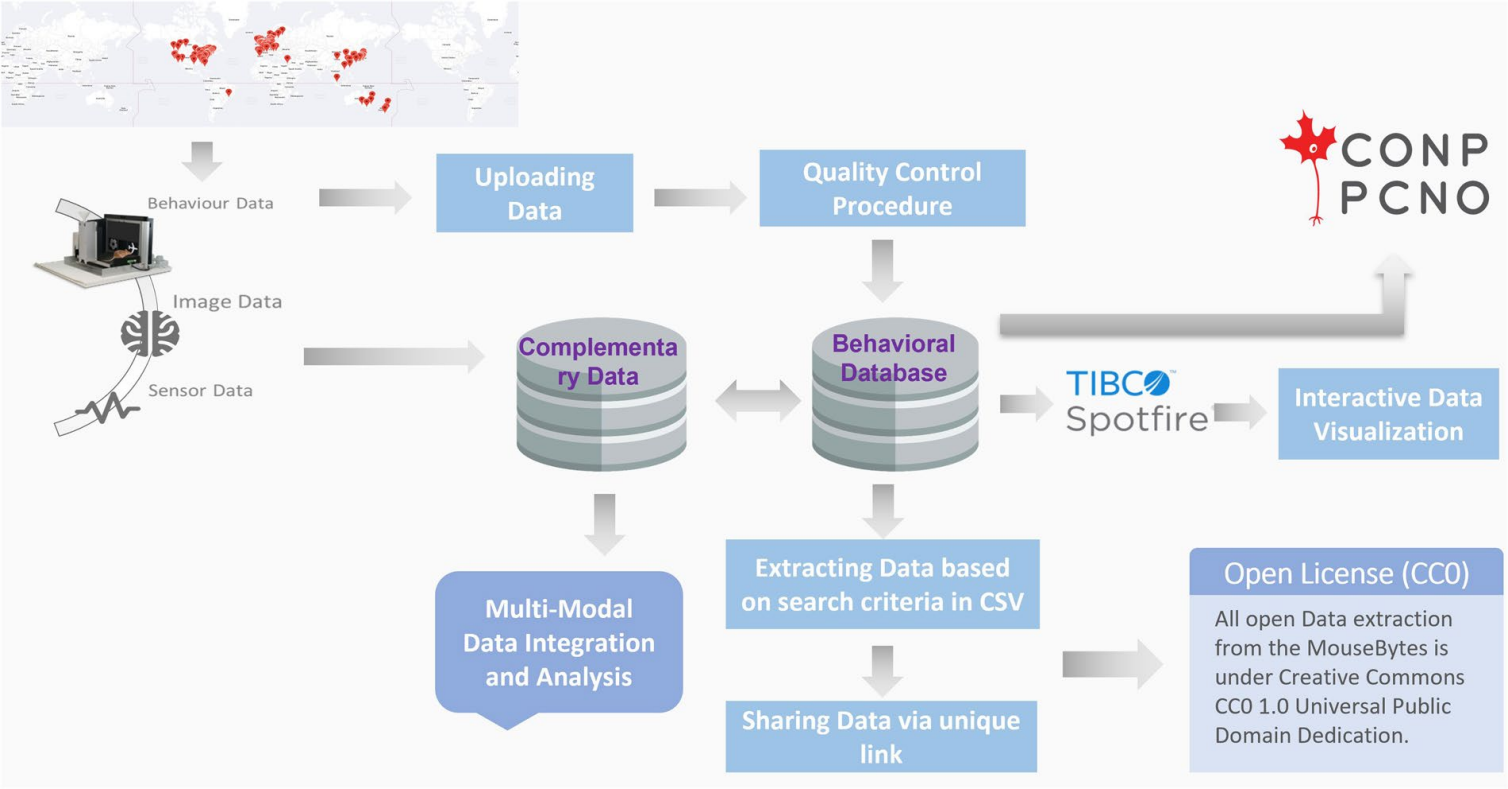
System overview.

MouseBytes employs advanced web technologies and is connected to a database of cognitive data obtained using touchscreen technology, allowing its use without any software installation and facilitating convenient collaboration, storage, sharing, and analysis of data. Figure 9 shows an overview of MouseBytes and the components that have been developed in this platform to make it Open Science and FAIR-compliant. Touchscreen users at different locations around the world collect data. These data are machine-generated and standardized and can be exported in XML (Extensible Markup Language) files using the Animal Behaviour Environment Test II (ABET II (https://lafayetteneuroscience.com/products/abetii-touch-screen-software)) in commercial systems. In this application, cognitive data can be exported in CSV and XML format. But, when users export the data in csv format, it limits the features that are exported and depends on the user to select these features. The xml files automatically have all features required to generate datasets and metadata. So, the csv option allows for customized data extraction, and the xml includes all the functions for a cognitive experiment.

Once data are uploaded to the database, the content of each file is checked against quality control rules specific for each cognitive test to make sure valid and accurate data are transferred to MouseBytes. This process is automated and incorporated into MouseBytes to handle errors that may occur due to software issues, human error, or rodent under-performance. In the next step, data in the database can be searched and queried based on criteria such as type of cognitive test, sex, age of animal, etc. A unique link can be generated for the extracted data and shared with other investigators. Open data in MouseBytes can also be visualized. Tibco Spotfire (https://www.tibco.com/products/tibco-spotfire), a prominent tool for data analysis and creating dashboards, was integrated to the MouseBytes. Other data modalities (e.g. fiber photometry, MRI Imaging, optogenetics, etc.) can be integrated with their corresponding touchscreen behavioral data through a unique link and functionalities developed in MouseBytes+ (https://mousebytes.ca/comp-search).

Finally, in order to make cognitive behavioral data more visible and transparent, we integrated MouseBytes data with the Canadian Open Neuroscience Platform (CONP (https://portal.conp.ca/)). Once a manuscript related to an experiment or dataset is published and assigned a DOI, its status is changed to public in MouseBytes, and it will be then integrated with CONP assuming that data will not be modified after being published. However, in a case that a user needs to make changes to an experiment (e.g., adding new data) after publishing the paper, an email will be automatically sent to the administrator, and a flag is triggered to inform the system to automatically re-run the query, and the most updated version of the dataset will be extracted, integrated, and synced to the CONP portal through the DataLad (https://www.datalad.org/) recommended by CONP. So, all public, published datasets in MouseBytes are also accessible via the CONP portal in CSV format^25,29,55^ .

**Fig. 10:**
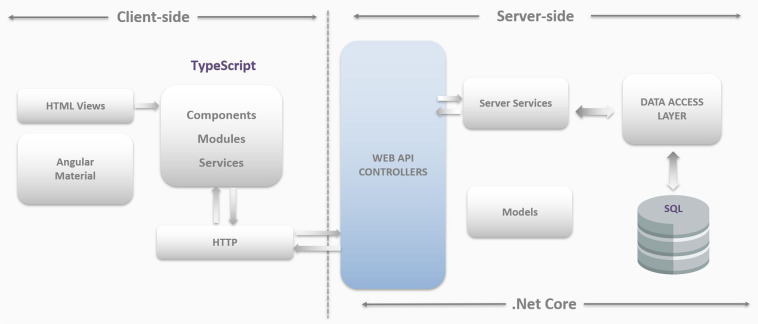
MouseBytes architecture.

## Data Availability

All the public datasets in MouseBytes are accessible through the Data Lab (https://mousebytes.ca/data-extraction) page described above. All the required metadata associated with each dataset are provided in the drop down menus to facilitate extracting the data. As part of the Data Citation Implementation Pilot (DCIP) project, a practical roadmap was developed to implement data citation for scholarly data repositories into three phases of required, recommended and optional^26^. To be compatible with the required DCIP principles, we registered the metadata with Datacite (https://datacite.org/), and generated the DOIs for all the public datasets in both MouseBytes and MouseBytes+. Such unique and persistent links were provided in the Search page for MouseBytes (https://mousebytes.ca/search-experiment) and in the Repositories page (https://mousebytes.ca/comp-search?showall=true) for MouseBytes+ which provides full access to the public datasets along with their metadata in CSV format. In addition to cognitive data, other data modalities such as MRI data and Fiber Photometry data correlated with behavioral analysis are hosted in the MouseBytes+ (https://mousebytes.ca/comp-search) where a unique link is assigned to each dataset to simplify data access and sharing provided that the status of the repository is public. The list of all the datasets in MouseBytes+ can be found in MouseBytes+ Repositories (https://mousebytes.ca/comp-search?showall=true) page.

## Code availability

Figure 10 shows the architecture of MouseBytes. This platform is based on ASP.NET WEB API framework in which Angular 5.0 was used in the client side, and .NET Core 2.0 was used in the server side. Angular is a Javascript framework consisting of HTML templates, TypeScript components, modules, and services. The client communicates with the server through the Angular services to call the API endpoints in the server. In the server side, there are API controllers which are used by client services for communicating data, and they handle the business logic of the application along with the services and models. All of these elements are connected to the database (Microsoft SQL Server 2017) through the data access layer. The code repository for MouseBytes is available in Github (https://github.com/Rodent-Cognition-Core/CBAS) under the GPL 3.0 license.
